# The ketosis-prone diabetes diagnosis dilemma-a case report

**DOI:** 10.1186/1758-5996-7-S1-A104

**Published:** 2015-11-11

**Authors:** Camila Viecceli, Sheila Piccoli Garcia, Thizá Massaia Londero, Ana Marina da Silva Moreira, Iuri Martin Goemann, Gustavo da Fonseca Cipriani, Themis Zelmanovitz

**Affiliations:** 1 HCPA, Porto Alegre, Brazil

## Background

Ketosis-prone diabetes (KPD) comprises diabetic ketoacidosis (DKA) without phenotypic characteristics of type 1 diabetes mellitus (T1D).

## Objective

Discussion of KPD diagnosis through a case presentation.

## Materials and methods

Clinical report and literature review.

## Results

A 16-year-old African American male was brought to emergency with abdominal pain, vomiting, lack of appetite and loss of 13kg (regular weight 152Kg, BMI 42.5kg/m^2^). Evaluation detected dehydration, blood glucose of 550mg/dL, acidosis and ketonemia. There was no previous diagnosis of diabetes. He received management for DKA, with fluid therapy and regular insulin via continuous intravenous infusion (around 100-140UI/day) for 4 days. Subcutaneous insulin regimen with NPH and regular at meals was initiated after correction of acidosis. He kept capillary glycemia around 250mg/dL despite increasing doses of insulin and exclusion of other pathologies. Detectable C-peptide (1.32; 0.78-5.19ng/mL), negative glutamic acid decarboxylase antibodies (anti-GAD), along with laboratory tests done a year ago showing fasting plasma glucose of 117mg/dL and glycated hemoglobin (A1c) of 6.2%, justified starting metformin 850mg/day. Patient evolved with marked improvement of glycemic control and was discharged. He returned at the diabetes clinic after 1 month, bringing his self-monitoring showing capillary blood glucose between 60-130mg/dL and A1c of 9.3%. Insulin was gradually reduced and metformin increased to maximum dose, leading to KPD hypothesis. Patient is currently receiving outpatient treatment and waiting result of HLA assessment.

## Discussion

KPD presents with classical symptoms of DKA, but majority of patients are overweight/obese and middle-aged Afro-American males. Incidence is increasing in younger patients. KPD is classified into four Aβ groups, based on the presence or absence of autoimmunity (A+; A-) and pancreatic β-cell functional reserve (β+; β-). Negative anti-GAD and high C-peptide level classify the patient described above as A-β+ subgroup, which is characterized by DKA findings with clinical features of type 2 diabetes. Forty percent of these patients achieve insulin independence and glycemic control with oral agents only. Insulin withdrawal can be longer than 10-14 weeks.

## Concluison

KPD should be investigated in young obese patients presenting with DKA, since treatment and follow up differs from T1D. Reversible β-cell dysfunction and remission to near normoglycemia is possible.

